# An Interactive Care System Based on a Depth Image and EEG for Aged Patients with Dementia

**DOI:** 10.1155/2017/4128183

**Published:** 2017-07-18

**Authors:** Xin Dang, Bingbing Kang, Xuyang Liu, Guangyu Cui

**Affiliations:** School of Computer Science and Software Engineering, Tianjin Polytechnic University, Tianjin 300387, China

## Abstract

Due to the limitations of the body movement and functional decline of the aged with dementia, they can hardly make an efficient communication with nurses by language and gesture language like a normal person. In order to improve the efficiency in the healthcare communication, an intelligent interactive care system is proposed in this paper based on a multimodal deep neural network (DNN). The input vector of the DNN includes motion and mental features and was extracted from a depth image and electroencephalogram that were acquired by Kinect and OpenBCI, respectively. Experimental results show that the proposed algorithm simplified the process of the recognition and achieved 96.5% and 96.4%, respectively, for the shuffled dataset and 90.9% and 92.6%, respectively, for the continuous dataset in terms of accuracy and recall rate.

## 1. Introduction

The expected growth of the older adult population in China over the next 30 years will have an unprecedented impact on the healthcare system, especially in terms of supply and demand for healthcare workers. Moreover, the elders are always short of self-care ability and require manual care. Nurses have heavy working burden, especially when they are taking care of the high-risk and 24-hour guardian patients. In addition, the rehabilitation training always depends on the experienced therapist. In fact, the shortage of practitioners of nursing and rehabilitation is serious. Therefore, the development of cheap and efficient intelligent care equipment is a significant way to solve these problems.

With the development of automatic rollover, efficient bedsore care beds appeared firstly and got a social positive assessment in 2007. This research achieves some good results on the smart rollover and bedsore care facility aspects. However, due to the high cost and complexity in a hardware system and with the lack of automation, those techniques are still in the lab.

Another limitation of those healthcare facilities is the low efficiency of the communication between the equipment and elders. The help requirement of the elders always includes feeding, going to the toilet, rehabilitation and massage treatments, or chat with someone. However, there are some difficult states that are not well settled for traditional human-machine interface technologies, such as accent pronunciation, weak sound, and difficulty in moving. Thus, improving the efficiency and confidence of the elders in their interaction with healthcare facilities becomes one of the most important research topics, in favor of both self-care and rehabilitation and reducing the burden on their children.

With the increasing use of portable computing and communication devices, researchers try to use smartphones or tablet in improving the convenience in the healthcare interactions. However, because of the declining vision, dry fingers, complex operation process, and high-power consumption, these devices will be abandoned after a period of time. Furthermore, the development of somatosensory technology provides a superior interaction experience in the medical rehabilitation. Some low-cost somatosensory sensors provide a high practical interaction system by gesture recognition and speech recognition algorithm and are widely used in the active sports therapy and rehabilitation of patients [1–4].

For the neuropsychology rehabilitation service, Chang et al. developed a rehabilitation system, Kinect's Kinempt, based on the cognitive impairment [1]. This system utilized fast rehabilitation simulation according to the patient rehabilitation in a pizza shop catering. Task instructions in the form of real-time video get feedback on users. Two patients measured by excitation in the system voice performed pizza topping choices. If the action of the wrist extracted by Kinect matches the eating action, encourage voice will be provided; otherwise, an error message is displayed. The successful patient training rate of the rehabilitation treatment is between 20% and 60% for the absence of the intervention system. The success rate of patient training is over 90% with the rehabilitation treatment intervention. In order to slow down mental illness, for example, Alzheimer's disease (senile dementia), Chiang et al. developed an interactive game for elderly that can improve somatosensory cognitive function [5]. To complete the gaming experience by a plurality of operational tasks within the prescribed time, the system provides an evaluation procedure. The experiences using a Kinect video game are carried out for around 4 weeks. The reaction rate has improved as well as the hand-eye coordination.

For the physical rehabilitation service, Chang et al. developed a system based on the Kinect Kinerehab [6]. The athlete patients are under the rehabilitation guidance. The system uses Kinect motion image processing techniques to extract information about the patient. The patients are required to carry some rehabilitation operations such as consistently moving and lateral rising of the arms. The patient's joint position will be drawn, and Kinect computer database matching precisely calculates the degree of action in place. The results are fed back onto the display device. Meanwhile, patients are encouraged to test the effects of rehabilitation. In the absence of an intervention system stage, the accuracy rate of tested results is low; in a systematic intervention phase, the accuracy rate of measurement was increased significantly.

For the balance therapy rehabilitation service, Lange et al. combine the virtual reality and video game technology and developed 3D models for spinal cord injury and traumatic brain injury patients with a balance rehabilitation training game, which applied Unity3D engine into the development platform to develop [7–9]. During the game, the system reflects an image with virtual human posture and movement state of patients. A Kinect sensor tracks objects in real time. Patients could adjust the balance of the shoulder, elbow, and other parts through one received feedback information. To achieve real-time interaction with virtual people, they improve the balance of perception. Patients can perform flexion and external rotation, and offsite training in subjects such as passively assisting the shoulder in the medical division complete the scheduled action.

For the occupational therapy rehabilitation service, Gama et al. designed a shoulder and elbow exercise rehabilitation training system [10] based on Kinect. In the rehabilitation process, patients maintain a high shoulder and arm movement was held side bottom. If the measured results maintain the correct body posture, the system interface information bar will display a real-time activity of the joint. Else, if the action fails to meet the requirements, the system corrects the action by extracting a depth image captured by a Kinect sensor and calculating shoulder and elbow angles (angle of the two connection lines shoulder-elbow and shoulder-hip). Redress ways include correcting the curved portion of the elbow and plane deviated. When the angle between a normal vector of the shoulder-hand and a crown vector connecting the node plane is not equal to 90°, the system will show error and suggest that elbow movements of patients deviate from the crown plane; if the vector sum of the shoulder-elbow and elbow-hand is not equal to that of the shoulder-hand connection vector, the system will show error and point that a patient has a bent portion on the elbow.

For the interaction assistance in rehabilitation, Luo et al. use a Kinect sensor that recognizes a target after extracting patient gestures. The recognition result was converted into control commands. The commands are transferred to patients' wheelchairs through the internet to control the chairs intelligently, to help lower limb movement disorder patients in the rehabilitation life.

In this paper, we proposed a novel system based on the multimodal deep neural networks for the patient with dementia with special needs. The input features of the networks are extracted based on the depth image sensor (Kinect) and electroencephalogram sensor (OpenBCI). The output layer will result in a type recognition of the patient's help requirement.

This paper is organized as follows.  Section 2 describes the collection of the training dataset, system flowchart, and the feature extraction.  Section 3 gives the performance help requirement a recognition algorithm and then compares it with other similar methods. Finally, Section 5 gives a practical application of this system and future research directions.

## 2. Data and Methods

### 2.1. Dataset Collection

In the current investigation, 15 elderly patients aged 55–70 with limited mobility and vocality from Tianjin nursing home were used as study subjects. All the participants are not in good health conditions; 8 of them are diagnosed as having dementia and 7 are diagnosed as having mild dementia. For each participant, 3-hour depth image and EEG data are recorded during their activity time with a continuous close-and-open eye movement as a synchronization pulse in the beginning of the data record. All the features are extracted from each event record and labeled with the types of the requirement by the nurses. After all, 1800 samples are packed into the dataset with 10 groups, for the cross-validation method in the neural network training and evaluation. The training set is shown in Table 1.

### 2.2. Overview of the System

**Figure 1:**
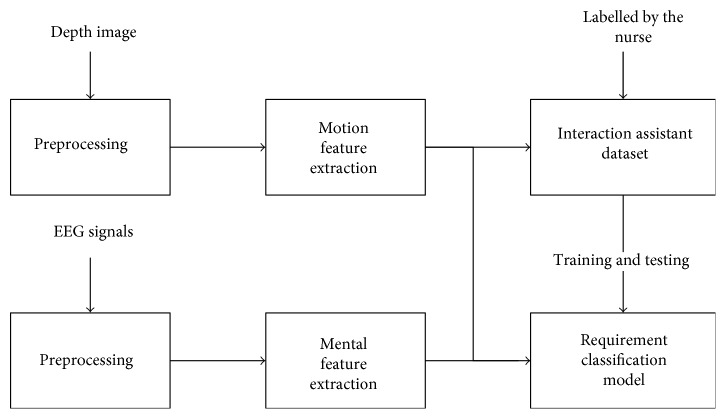
The system flowchart.

The system acquired the record of the user's motion and mental states based on depth image and electroencephalogram signals by the Kinect and OpenBCI sensors, respectively. The motion features are extracted form a depth image after preprocessing with a filter, skeletal point tracking, region division, and normalization. The mental features are extracted from electroencephalogram signals after preprocessing with a bandpass filter and normalization. All the records of the interaction requirement activity are labelled by the nurse and collected into the dataset; the classification models are then trained and tested by the dataset. The flowchart of proposed system is shown in Figure 1.

### 2.3. The Feature Extraction of a Depth Image

**Figure 2:**
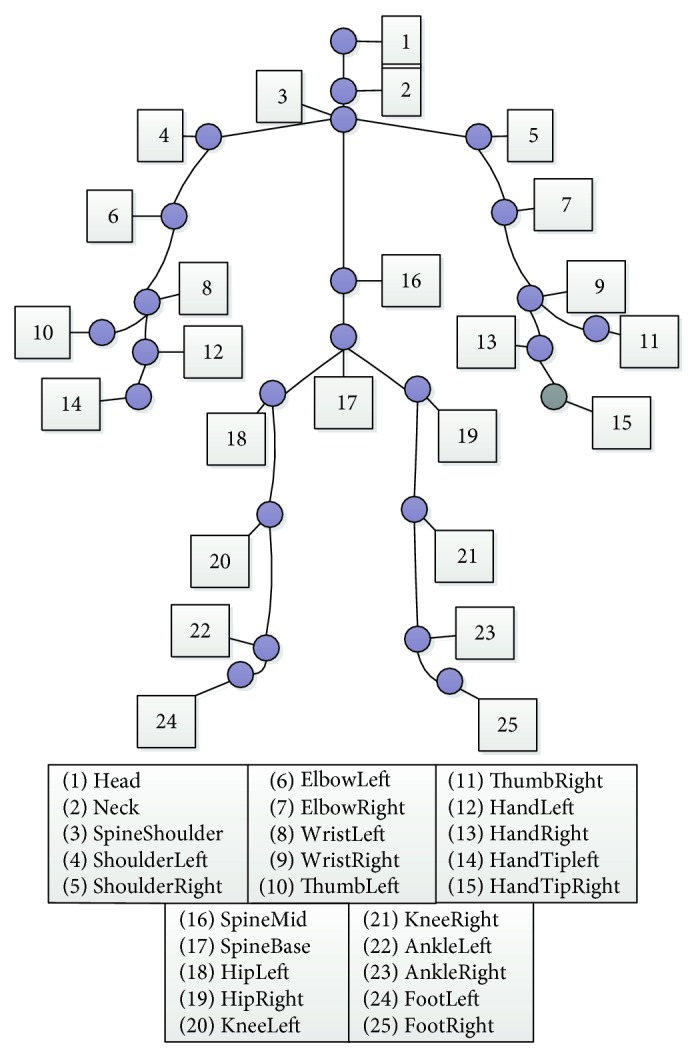
Skeleton tracking-based Kinect.

The system analyzes the users' gesture by extracting 20 skeletal points from the depth image with a Kinect sensor. The three-dimensional coordinates of all the skeletal points are calculated and normalized using the spine skeletal coordinate as the origin [11]. The skeletal points are shown in Figure 2. Six regions are divided as the input feature of the system; they are cervical area, left wrist, right wrist, crotch, left knee, and right knee. Preprocess algorithms calculate the motion feature with the relative distance and direction of the regions, which were calculated by the location of the region center and their corresponding direction. Many activities in the dataset occur in 2-3 seconds; thus, in order to extract the feature changing in the help interaction, a three-segmentation scheme is applied for each record.

### 2.4. The Feature Extraction of EEG Signal

**Figure 3:**
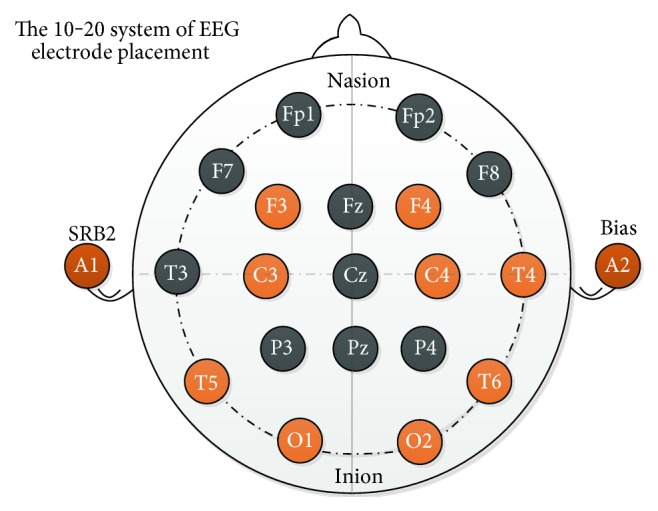
The electrode placement.

After the filtering process, then, this 8-dimensional data are put into the proposed system. For each channel, four band signals provided based on the frequency by the OpenBCI denoted *δ* (0.1–3 Hz), *θ* (4–7 Hz), *α* (8–12 Hz), and *β* (12–30 Hz). The electrode placement includes F3, F4, C3, C4, T5, T6, O1, and O2 as shown in Figure 3. Similar with those of the motion feature extraction, the mean and variance of three divisions of each activity record are calculated for the mental features.

## 3. Autoencoder and Classification Model

### 3.1. Autoencoder

The proposed algorithm consists of two parts: (1) sparse encoder—mainly used to study the character expression of the users' operation in healthcare, and (2) softmax category layer—recognizes the user's action through softmax based on the expression of sparse feature from the encoder in the hidden layer:

1. Sparse autoencoder and unsupervised learning method

An autoencoder (AE) is a three-layer neural network that contains a visible layer, hidden layer, and reconstruction layer. Unsupervised learning and backpropagation (BP) algorithm and output vector equal to the input are used in the reconstructed layer.

**Figure 4:**
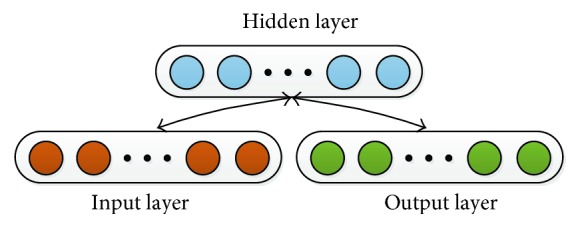
Schematic structure of an autoencoder.

A sparse autoencoder is a modified model as an encoder and obtains sparse characteristic through adding specific conditions to obtain the hidden layer in the training process [12–14]. We use the sigmoid function *f*(*a*) = 1/(1 + exp(−*a*)) as the activation function. The feature extraction process of an AE includes two stages as shown in Figure 4. In the first stage, the user's motion and mental features *x* were mapped to the hidden layer *z*. The second phase generates and outputs *y* by decoding and reconstructing *z*. These two stages can be formulated as follows:

(1) $$\begin{array}{c} z=f\left(W_{e}n+b_{e}\right),\\ y=f\left(W_{d}n+b_{d}\right). \end{array}$$

*W*_e_ and *W*_d_ are weight matrices of the encoder and the decoder, and *b*_e_ and *b*_d_ are the offset vectors. SAE training is used to minimize cost function *c*(*n*, *y*) by adjusting the parameters (*W*_e_, *W*_d_, *b*_e_, and *b*_d_) using the BP algorithm.

(2) $$\begin{array}{c} \underset{W_{e},W_{d},b_{e},b_{d}}{\arg\;\min\;}\left[c\left(n,y\right)\right], \end{array}$$

where *c*(*n*, *y*) denotes the error between the reconstructed layer and visible layer.

While input features are reconstructed in an output layer, high-level potential features related to users' requirement can be extracted in the hidden layer; the parameters of the hidden layer can be served as inherent characteristics of users' requirement. Hence, an *n*-dimensional input feature vector of the user's activity can be converted to *h*-dimensional potential features of users' requirement.

The potential features *e*(*x*) in the hidden layer can express input vectors in a compression method. When removing redundant relationship of input vectors after adding restrictions, *e*(*x*) can explore the significant cross-correlation structure. This feature making the instance correspond to the input feature vector can be represented as a sparse mixture. Redundancy of joint distribution of any two features is compressed minimally in an SAE model. This model can ensure the robustness of the system when the input feature vectors have some damages. Therefore, this structure of the encoder achieves great success in the varieties of mode feature extraction. SAE cost function is defined as follows:

(3) $$\begin{array}{c} c\left(n,y\right)=\frac{1}{2q}\overset{q}{\underset{i=1}{\sum }}{\left\|n^{\left(i\right)}-y^{\left(i\right)}\right\|}_{2}^{2}+\alpha \overset{h}{\underset{i=1}{\sum }}KL\left(\rho \left\|{\hat{\rho }}_{i}\right.\right). \end{array}$$

The first term is the average of differences among *q* input vectors, and the second is the sparse penalty term generated by *h* neurons in the hidden layer. The constant *α* denotes the control coefficient of sparse penalty. The KL divergence of two random variables in constraint entry can be formulated as follows:

(4) $$\begin{array}{c} KL\left(\rho \left\|{\hat{\rho }}_{i}\right.\right)=\rho \log\frac{\rho }{{\hat{\rho }}_{i}}+\left(1-\rho \right)\log\frac{1-\rho }{1-{\hat{\rho }}_{i}}, \end{array}$$

where ρ^ i=1/q∑j=1qzinj is the activity of *j* neuron and *ρ* is the sparse parameter.

(2) Stacked autoencoder and supervised learning method

A stacked autoencoder neural network is composed of multilayer sparse encoders. The output of the previous layer is the input of the subsequent layer. Characteristic expression of the last hidden layer will be put into a softmax classifier through the types of help requirement. The training process includes a pretraining stage and refine stage [2, 12, 15, 16]:

- Pretraining stage

**Figure 5:**
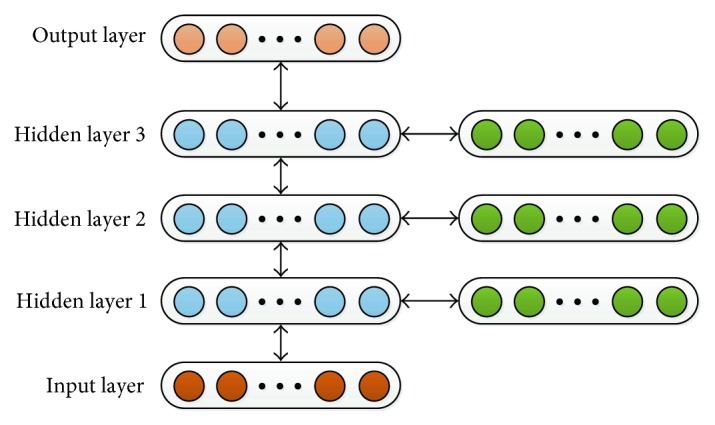
Stacked autoencoders.

A greedy scheme is applied in the training of SAE, the training method, as shown in Figure 5. The hidden layer of the *i*th autoencoder layer is the input of the next autoencoder.

(b) Refine stage

The proposed algorithm took the last hidden layer of the third autoencoder as the output layer of supervised training, by using the backpropagation algorithm. Each output unit has a corresponding label. Then, weights in the network are refined by the BP algorithm using the labelled data.

**Figure 6:**
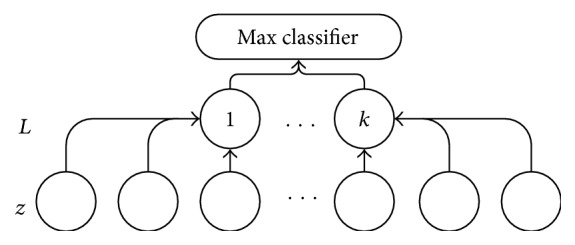
Softmax classification model.

The softmax regression (SR) model used in the BP algorithm is shown in Figure 6. (*z*, *L*) is annotated training data in the model, where *z* is the expression of characteristic of the hidden layer and *L* is the classification label; users' activities are labeled from help requirement types from 1 to *k*.

The probability vector of the SR model *r*_*θ*_(*z*) can be formulated as follows:

(5) $$\begin{array}{c} r_{\theta }\left(z\right)=\left[\begin{array}{c} \begin{array}{c} p\left(l=1\right)\\ \ldots \end{array}\\ p\left(l=m\right) \end{array}\right]=\frac{1}{\sum_{i=1}^{m}e^{\theta_{i}^{t}z}}\left[\begin{array}{c} e^{\theta_{i}^{t}z}\\ \ldots \\ e^{\theta_{m}^{t}z} \end{array}\right], \end{array}$$

where *θ* = [*θ*_1_^*t*^,   …  , *θ*_*m*_^*t*^]. *p*(*l* = *m*) is the predicted probabilities of the user's *m*th class label for each value of *m* = 1,   …  , *k*. The output of the SR model will be the help requirement class label with the highest probability results.

Thus, the cost function of the SR model can be formulated as follows:

(6) $$\begin{array}{c} J\left(\theta \right)=\frac{1}{q}\left[\overset{q}{\underset{i=1}{\sum }}\overset{m}{\underset{j=1}{\sum }}1\left\{l=j\right\}\;\log\frac{e^{\theta_{i}^{T}z}}{\sum_{j=1}^{m}e^{\theta_{i}^{T}z}}\right], \end{array}$$

where *l{.}* is the indicator function, that is, *l {true} =* 1 and *l{false} =* 0. *θ* denotes all the parameters of our model and ensures that the cost function *J*(*θ*) is minimum.

(3) SAE-based multimodal classification

Due to the health condition, the aged always have an accent, weak sound, and limited body movement. For these situations, by imitating the workflow of a nurse in the healthcare, we extracted the patient's gestures and mental state from their body movement and EEG features. Furthermore, three types of the SAE-based multimodal models are proposed. And the details of them are shown as follows.

**Figure 7:**
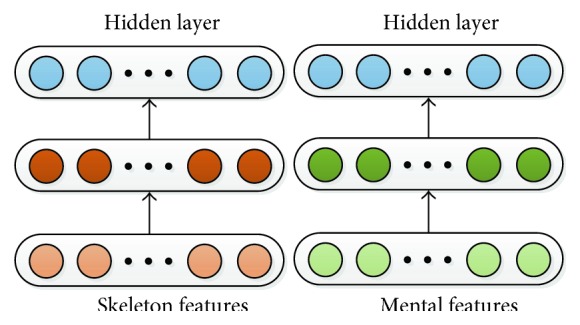
Single-modal classification model based on SAE.

For the first type, the SAE model is pretrained and refined with the patient's motion and EEG features, respectively. The SAE network structures are shown in Figure 7.

**Figure 8:**
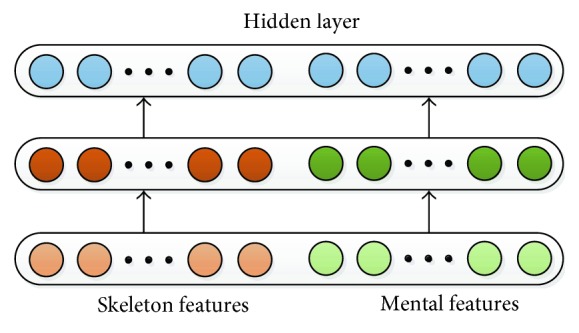
Multimodal classification model based on SAE.

For the second type, the input features of the motion and EEG are integrated at the first layer and pretrained with the integrated features in each hidden layer. The output layer is calculated by the integrated hidden layer. In the refine stage, the model is trained by the integrated features and labeled data. The network structure is shown in Figure 8.

**Figure 9:**
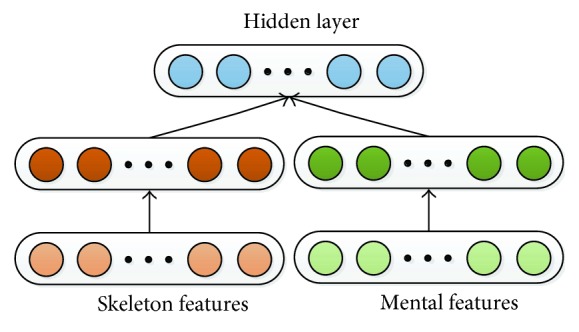
Multimodal classification model based on SAE.

For the third type, the input features of the motion and EEG are pretrained, respectively, at the second layer and integrated in the third layer. The output layer is calculated by the integrated hidden layer. In the refine stage, the model is trained by the two-modal features and labeled data. The network structure is shown in Figure 9.

Those three architectures are trying to imitate the workflow of the health nurses. The first model predicts the patient's requirement with the skeletal and EEG features, respectively; the second model predicts the patient's requirement with the integrated skeletal and EEG features with limited use of the deep neural network as a high-level feature extraction; and the third model extracts a high-level feature in each hidden layer and integrates the high-level feature of the two-modal signals, then completes the classification by the SR model.

## 4. Experimental Results

SAE is built and trained based on the MATLAB Deep Learning Toolbox, which includes (1) SAE trained by the skeletal features, (2) SAE trained by EEG, and (3) SAE trained by the integrated data of both. All of the three SAEs contain three hidden layers.

For single-modal networks, the hidden layer size are set as 80 and the input sizes of the skeletal modal and EEG modal were set as 200 and 80, respectively. For a multimodal network, hidden layer size was set as 80; the input sizes of the skeletal modal and EEG modal were set as 200 and 80, respectively; and the size of the hidden layer was set as 160.

In the evaluation, the performance of a multiple recognition model based on SAE has been compared: classification based on skeletal features (skeleton), classification based on EEG feature (EEG), classification based on skeletal and EEG features (skeleton-EEG) (see Figure 6), and integration of two-modal features in hidden layers (integrated) (see Figure 7). Furthermore, as a comparison, the DTW method [17] is added into the evaluation.

In the evaluation, the accuracy *P*, the recall rate *R*, F1, and time consumption are used as the indicators; they are calculated as follows:

(7) $$\begin{array}{c} P=\frac{A_{g}}{A_{g}+A_{n}}\times 100;\\ R=\frac{A_{g}}{A_{g}+A_{n}}\times 100;\\ F1=\frac{2\cdot P\cdot R}{P+R}, \end{array}$$

where *A*_*g*_ identified as the classification is distinguished correctly, *A*_*n*_ is misrecognition, and *N*_*g*_ means the recognition that the patient does nothing but was recognized with other classes.

The proposed methods are tested on two types of dataset:

1. All classes of the help requirement record are extracted and shuffled into a dataset. The feature and label of each record are extracted and collected as the training data and test data sets.
2. All raw data are divided into record samples by the frame. The frame length and frameshift are 3 seconds and 50%, respectively. The feature and label of each sample are extracted and collected as the training data and test data sets.

For the first type of dataset, the experiment results of the methods are shown in Table 2. Compared with that of three single-modal methods, the accuracy of the multimodal models achieved 5%–7% improvement under the same level of recall ratio. The integrated method showed the best accuracy and recall rate. The results of the skeleton-EEG method have a large improvement than those of the EEG or skeleton method in terms of accuracy and recall rate. The results of the skeleton method are little worse than those of the EEG-based model. It is suggested that the motion feature is insufficient for the activity recognition of the patient with dementia. However, skeleton features in the skeleton-EEG method and integrated method improve the performance in terms of both accuracy and recall rate. It is suggested that the multimodal method is better on exploring the characteristics of the physical and psychological activities in healthcare interaction for the dementia patients.

For the second type of dataset, the results are shown in Table 3. The performance is decreased in each term due to the frame division of the dataset. Compared to the traditional DWT algorithm, the SAE models achieved higher recognition accuracy, despite that the single-modal models resulted in a little bit lower recall rate. Two multimodal models showed good performance both in the accuracy and in the recall rate. It is suggested that the multimodal model results in a significant improvement of the recall rate with the same level of the accuracy. The integrated model further increased the recall rate for the continuous input samples.

## 5. Conclusions

Healthcare and nursing robot achieve wide attention in recent years [18–21]. Somatosensory technology has been introduced into the activity recognition and healthcare interaction. However, due to the limited body movement of the patients, the traditional single-modal method is unsuitable to settle this interaction problem. On the other hand, with the development of the deep neural network technology, the depth model is applied as a high-level feature extractor and multimodal feature integrator. In order to develop an efficient and convenient interaction assistant system for nurses and patients with dementia, two novel multimodal SAE frameworks are proposed in this paper based on motion and mental features. The motion and mental features are extracted after the preprocessing of depth image and EEG signals acquired from Kinect and OpenBCI, respectively. The proposed algorithms simplify the acquisition and data processing under high action recognition ratio compared with the traditional DTW method and performed better in terms of both accuracy and recall rate.

## Figures and Tables

**Table 1:** The label and features in the training set.

The types of requirement	Number of samples	Movement and mental features
Sleeping	192	Hand near the cheek and tired
Standing	198	Open arms and vibrant
Walking	202	Knee up and intent
Drinking	208	Hand near the mouth and thirsty
Eating	193	Hand near the mouth and hungry
Defecation	207	Head movement and defecating urgently
Urination	215	Head movement and urinating urgently
Calling the doctor	185	Hand movement and urgent
Nothing	200	None

**Table 2:** Results of classification of the methods on the shuffled dataset.

Classifier	Accuracy (%)	Recall rate (%)	F1 measure	Time (s)
EEG	94.2	92.8	93.7	0.01
Skeleton	93.8	93.4	93.8	0.01
Skeleton-EEG	94.7	94.6	94.2	0.01
Integrated	96.5	96.4	96.2	0.01

**Table 3:** Results of classification of the methods on the continuous dataset.

Classifier	Accuracy (%)	Recall rate (%)	F1 measure	Time (s)
DTW [17]	84.2	90.0	89.6	0.035
EEG	90.1	87.6	87.7	0.01
Skeleton	89.3	86.4	86.9	0.01
Skeleton-EEG	90.7	90.4	89.5	0.01
Integrated	90.9	92.6	91.3	0.01
